# Immunotherapeutic Approaches in Malignant Pleural Mesothelioma

**DOI:** 10.3390/cancers13112793

**Published:** 2021-06-04

**Authors:** Rita Terenziani, Silvia Zoppi, Claudia Fumarola, Roberta Alfieri, Mara Bonelli

**Affiliations:** Department of Medicine and Surgery, University of Parma, 43126 Parma, Italy; rita.terenziani@unipr.it (R.T.); silvia.zoppi@unipr.it (S.Z.); claudia.fumarola@unipr.it (C.F.)

**Keywords:** immunotherapy, immune checkpoint inhibitors, PD-1/PD-L1, CTLA-4, VISTA, malignant pleural mesothelioma

## Abstract

**Simple Summary:**

Immune checkpoint inhibitors (ICIs) have emerged as a very promising therapeutic option for the treatment of many difficult-to-treat cancers and a number of clinical trials have explored their efficacy in malignant pleural mesothelioma patients. ICIs were initially evaluated in the salvage setting, resulting in a modest activity, not superior to chemotherapy. However, in the last year the combination of nivolumab and ipilimumab as first line treatment has proved a superior efficacy compared to chemotherapy especially in the non-epithelioid subtype, obtaining the FDA approval in October 2020. Encouraging results are also emerging from other immunological approaches that take advantage of tumor-specific antigens, such as advanced cell-based therapies with the CAR-T cells and tumor vaccines.

**Abstract:**

Malignant pleural mesothelioma (MPM) is a rare and aggressive malignant disease affecting the mesothelium, commonly associated to asbestos exposure. The current therapeutic actions, based on cisplatin/pemetrexed treatment, are limited due to the late stage at which most patients are diagnosed and to the intrinsic chemo-resistance of the tumor. Another relevant point is the absence of approved therapies in the second line setting following progression of MPM after chemotherapy. Considering the poor prognosis of the disease and the fact that the incidence of this tumor is expected to increase in the next decade, novel therapeutic approaches are urgently needed. In the last few years, several studies have investigated the efficacy and safety of immune-checkpoint inhibitors (ICIs) in the treatment of unresectable advanced MPM, and a number of trials with immunotherapeutic agents are ongoing in both first line and second line settings. In this review, we describe the most promising emerging immunotherapy treatments for MPM (ICIs, engineered T cells to express chimeric antigen receptors (CARs), dendritic cells (DCs) vaccines), focusing on the biological and immunological features of this tumor as well as on the issues surrounding clinical trial design.

## 1. Introduction

Malignant pleural mesothelioma (MPM) development is widely known to be related to asbestos exposure. Investigations of the role of tumor microenvironment (TME) suggested that the massive interaction between the immune infiltrate and the asbestos-damaged mesothelial cells may have an important role in determining tumor development and progression [1]. The chronic inflammation of the pleura in response to asbestos fibers produces a typical TME, the composition of which might affect the prognosis. Indeed, it has been described that the presence of immunosuppressive cells such as regulatory T cells (Treg_s_), macrophages, and myeloid-derived suppressor cells (MDSCs) prompts MPM progression [2,3]. A chronic exposure to asbestos of HTLV-1 immortalized human suppressive T cell line MT-2, enhanced their Treg functions via cell–cell contact and increased the production of suppressive cytokines, such as IL-10 and TGFβ [4], indicating that asbestos exposure may reduce the anti-cancer immunity. This unique TME and the prevalence of an immunosuppressive signature during MPM progression provide the rationale for the use of immune checkpoint inhibitors (ICIs) as a treatment option for MPM patients. On the other hand, high levels of CD8^+^ tumor infiltrating lymphocytes (TILs) have been positively correlated with tumor regression and improved survival [5].

Currently, the existing therapeutic actions against MPM are limited due to the late stage at which most patients are diagnosed and to the intrinsic chemo-resistance of the tumor. Indeed, only few patients are eligible for the trimodality therapy, including surgery, chemotherapy, and radiotherapy. The recommended systemic therapy for unresectable MPM patients is cisplatin/pemetrexed treatment (regimen that in some conditions may be improved by the addition of bevacizumab) with still limited clinical benefit, short-term regression, and local tumor relapse. In addition, differently from other solid tumors, the current clinical guidelines do not recommend biological targeted therapy for MPM patients, mainly because of poor target definition and the lack of known oncogenic driver alterations [6]. Another relevant point is the absence of approved therapies in the second line setting following progression of MPM after chemotherapy. In this setting, therapies with ICIs, which disrupt mechanisms of immune evasion by tumor cells, are under evaluation.

**Figure 1:**
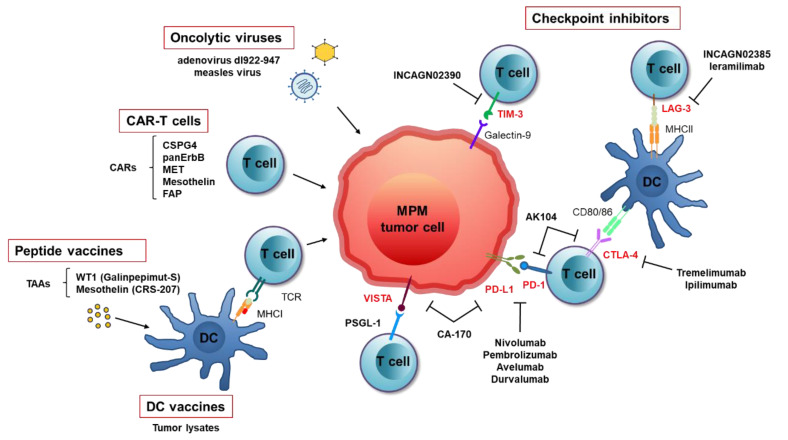
Immunotherapy approaches in MPM. CARs, chimeric antigen receptors; CSPG4, chondroitin sulfate proteoglycan 4; CTLA-4, cytotoxic T lymphocyte protein 4; DC, dendritic cell; FAP, fibroblast activation protein; LAG-3, lymphocyte activation gene 3; MHC, major histocompatibility complex; MPM, malignant pleural mesothelioma; PD-1, programmed death 1; PD-L1, programmed death ligand 1; PSGL-1, P-selectin glycoprotein ligand 1; TAAs, tumor-associated antigens; TCR, T cell receptor; TIM-3, T cell immunoglobulin 3; WT1, Wilms’ tumor 1.

Immune checkpoints have shown promise as therapeutic target in various types of tumor and drugs for immune checkpoint blockade entered the clinic as a standard of care for non-small cell lung cancer (NSCLC) and other solid tumors [7]. A number of ICIs have been tested to make a step forward in MPM treatment; among them, antibodies against cytotoxic T-lymphocyte associated antigen 4 (CTLA-4) and programmed death 1 (PD-1)/programmed death ligand 1 (PD-L1) are the most studied for MPM, as it has been reported that both CTLA-4 and PD-L1 expression correlate with a worse prognosis [8,9]. In the last few years, other immune checkpoints have been considered as possible mediators of immune escape by tumor cells; among them, LAG-3, TIM-3 and VISTA have been identified as possible targets in MPM patients [10,11]. In addition to immune ICIs, a number of immunotherapeutic approaches are under investigation for MPM, including immunotherapy based on chimeric antigen receptor (CAR) T cells and therapeutic vaccination strategies (Figure 1).

## 2. Immune Checkpoint Inhibitors

### 2.1. CTLA-4

CTLA-4 was one of the first immune suppressive receptors showing an inhibitory effect on T cells responses [12]. As a member of the CD28/B7 immunoglobulin superfamily, it is constitutively expressed on Treg cells and, to a lower extent, also on antigen presenting cells (APC), granulocytes, and activated effector T cells [12,13]. CTLA-4 binds to CD80 and CD86, thus interfering with CD28 co-stimulation and reducing the amplitude of T cell response [14,15]. In several murine mesothelioma models blockade of immune suppressive CTLA-4 resulted in therapeutic effects when administered alone, or when combined with chemotherapy or radiation [16,17,18].

When anti-CTLA-4 was administered alone in mesothelioma-bearing mice, it inhibited tumor growth and increased overall survival. The therapeutic effect was more evident, with complete tumor regression, when the anti-CTLA-4 agent was associated with inhibitors of OX40 (TNF receptor mainly expressed on T lymphocytes), efficaciously reducing Treg_s_ inside the tumor and increasing the activation and proliferation of CD8^+^ TILs [17]. The therapeutic effect became even more evident when the anti-CTLA-4 agent was administered between cycles of chemotherapy (cisplatin) and especially at the initial stage of tumor growth. Particularly, the effect was associated with an inhibition of cell repopulation and an increase in TILs, thus suggesting that inhibiting CTLA-4 improves the efficacy of chemotherapy [18]. Moreover, the association of anti-CTLA-4 agents with gemcitabine in a murine mesothelioma model, proved to have a synergistic effect against tumor growth. Interestingly, when mice were re-inoculated with tumor cells, about 93% of them were completely resistant to tumor rechallenge, thus suggesting that the combination induced immunological memory and therefore durable responses [19]. These studies suggest that administration timing is a crucial aspect for maximizing the efficacy: The anti-CTLA-4 agent should be administrated sequentially after every cycle of cisplatin, and concurrently when associated with gemcitabine [18,19].

To date, anti-CTLA-4 agents administered alone have shown clinical activity only in three studies. Initially, the open-label, single arm, phase II studies MESOT-TREM gave encouraging results. In the first one (NCT01649024), tremelimumab, a fully human monoclonal anti-CTLA-4 antibody, was administered at 15 mg/kg intravenously every 90 days. At the end of the study, no patient had a complete response (CR), two achieved a partial response (PR) and seven others achieved disease control (DC) with a median progression-free survival (PFS) of 12.4 months (Table 1). In the subsequent MESOT-TREM 2012 study (NCT01655888), tremelimumab dosage was reduced and the treatment schedule intensified: The 29 s line MPM patients were administered with 10 mg/kg once every four weeks for six doses and then every 12 weeks until disease progression or sever toxicity. At the end of the study, 52% of patients showed DC, with a median duration of 10.9 months [20,21].

Based on these results, a larger double-blind, placebo-controlled phase IIb trial, the DETERMINE study (NCT01843374), was performed, involving 571 patients with unresectable pleural or peritoneal malignant mesothelioma who had progressed after one or two previous systemic treatments for advanced disease. Patients were randomized (2:1) to tremelimumab or placebo and received tremelimumab at 10 mg/kg or matching placebo every four weeks for seven doses as an induction treatment, then maintenance dosing every 12 weeks until treatment discontinuation criterion. Preliminary results highlighted that tremelimumab treatment did not significantly improve overall survival (OS) compared with placebo, nor was able to improve PFS, objective responses (OR) and DC compared to the placebo [22]. Notably, a higher proportion of patients in the tremelimumab group showed treatment-emergent adverse events that were grade 3 or worse and led to discontinuation of study treatment. This different outcome, as compared with MESO-TREM studies, could be explained considering that DETERMINE was a multi-centric study involving a higher number of patients. Additionally, the absence of a predictive marker for CTLA-4 blockade made difficult to identify patients more likely to respond [22].

### 2.2. Anti-PD-1/PD-L1

Another important target in immunotherapy is represented by the immune checkpoint PD-1/PD-L1 PD-1 is an inhibitory receptor expressed by activated T cells, B cells, natural killer (NK) cells and myeloid cells, which binds to PD-L1, a cell surface glycoprotein of the B7 family primarily expressed on antigen-presenting cells, activated T cells, and stromal or tumor-infiltrating immune cells. PD-1 binds also PD-L2, another member of the B7 family. Binding of PD-1 to PD-L1 results in the reduction of the downstream signaling in T cells and in the downregulation of T cell immune responses by elevating the threshold of T cell activation, while T cell proliferation is inhibited, and T cell apoptosis is enhanced [23].

The clinical success achieved by PD-1/PD-L1 targeting in several malignancies, such as melanoma and NSCLC among others, promoted the study of these agents also for MPM treatment. To date, several studies with anti-PD-L1 or PD-1 agents have been performed.

Nivolumab, a human monoclonal antibody targeting PD-1, was tested in two single arm phase II trials (NivoMes and MERIT) and in the MAPS2 trial in association with the anti-CTLA-4 antibody, ipilimumab [24,25,26]. The NivoMes trial (NCT02497508) involved 34 patients with recurrent malignant mesothelioma that were administered with nivolumab at 3 mg/kg two times a week until progression or toxicity. The study met its primary endpoint, with eight patients showing PR at 12 weeks and eight showing stable disease (SD), resulting in disease control rate (DCR) of 47% at 12 weeks. Additionally, in four patients with SD the tumor remained stable for more than six months. Adverse events of any grade occurred in 26% of patients, commonly fatigue and pruritus, while grades 3 and 4 adverse events, such as pneumonitis and gastrointestinal disorders, were reported in nine patients [24]. However, the PFS of only 2.6 months was quite disappointing. The second study (MERIT) enrolled 34 patients that received nivolumab at 240 mg intravenously every two weeks until progression or toxicity. The study results showed a 68% DCR and a PFS of 6.1 months [25]. These results were relevant enough to lead Japan to approve nivolumab as a second line treatment agent for mesothelioma. Lastly, the MAPS2 (NCT02716272) was a multicenter, randomized, non-comparative, open-label, phase II study of nivolumab or nivolumab-ipilimumab. Patients were randomized (1:1) to receive intravenous nivolumab (3 mg/kg) every two weeks, or intravenous nivolumab plus intravenous ipilimumab (1 mg/kg every six weeks), given until progression or toxicity. In spite of the encouraging results of a DCR of 40% and a low percentage of adverse effects, the study could not provide any relevant difference between the two regimens [26]. The association of nivolumab and ipilimumab has been also evaluated in the INITIATE study (NCT03048474), a prospective single-center, single arm, phase II trial conducted in 38 MPM patients who progressed after platinum-containing chemotherapy. Patients received nivolumab (240 mg every two weeks) plus ipilimumab (1 mg/kg every six weeks up to four times) for up to two years or until confirmed progression or toxicity. The results of the study were encouraging, with 10 (29%) patients showing PR and 13 (38%) patients showing SD. The most common adverse events were infusion-related reactions, skin disorders, and fatigue [27]. However, the results of MAPS2 and INITIATE studies are difficult to compare, and further studies with an enlarged number of patients are required to clarify the potential benefit of the association over the single agent treatment. Very recently, a multicenter phase I/II trial has been designed to evaluate the safety and feasibility of the neoadjuvant immune checkpoint blockade therapy against resectable MPM (NCT03918252). Patients with treatment-naive MPM will be randomized 1:1 and receive nivolumab (240 mg) every two weeks for three doses with or without one dose of ipilimumab (3 mg/kg). After the macroscopic complete resection, patients will receive optional adjuvant chemotherapy or/with radiation. Primary co-endpoints are safety and feasibility, with safety evaluated by adverse events and feasibility evaluated by a delay in surgery ≤ 24 days. The study is now open for enrollment.

In patients with unresectable and previously untreated MPM, the association of nivolumab plus ipilimumab has been tested as a first line therapy vs. chemotherapy, in a phase III open-label, multicenter, randomized trial, the CheckMate 743 (NCT02899299). A total of 605 participants with performance status of 0 or 1 were randomized 1:1 and were administrated with nivolumab (3 mg/kg once every two weeks) plus ipilimumab (1 mg/kg once every six weeks) or platinum plus pemetrexed chemotherapy (500 mg/m^2^ plus 75 mg/m^2^). The study met its primary endpoint at interim analysis with median OS of 18.1 months in the nivolumab + ipilimumab arm vs. 14.1 months in the chemotherapy arm; OS rates were 68% vs. 58% at one year and 41% vs. 27% at two years. Notably, the OS improvement in patients treated with the ICI combination was not related to tumor histology. Indeed, the OS observed after ICIs treatment was similar among epithelioid and non-epithelioid patients (median OS of 18.7 months and 18.1 months in the epithelioid and non-epithelioid subtypes, respectively), while the OS with chemotherapy differed substantially among groups (OS rate in non-epithelioid histology 63% vs. 32% at one year and 38% vs. 8% at two years), as it is known that non-epithelioid histotype tumors have a worse prognosis and are less sensitive to chemotherapy treatment. Therefore, especially in non-epithelioid subtypes the immunotherapy-based combination provided meaningful improvement in OS versus standard-of-care chemotherapy. The increase in OS, along with the favorable clinical benefit-risk profile led to the 2020 FDA approval as a standard of care for patients with previously untreated unresectable MPM [28,29].

Nivolumab as a single agent has also been tested in an ongoing placebo-controlled, randomized, multi-center phase III trial (CONFIRM, NCT03063450) on a total of 336 patients with pleural o peritoneal mesothelioma who have already received at least two prior lines of therapy. Patients have been randomized nivolumab: placebo (2:1) and treated with 240 mg of nivolumab or a saline placebo solution for 12 months of treatment. The primary endpoints of the study were OS and PFS (modified RECIST or RECIST 1.1) [30]. Preliminary data from this study showed the safety of the treatment and proved that nivolumab improved both PFS rates at 12 months of treatment (14.5 in the nivolumab arm vs. 4.9 for placebo) and OS (9.2 months vs. 6.6). The significant clinical benefits were more evident for the epithelioid subtype [31].

Combination of nivolumab and chemotherapy is also being studied, based on the hypothesis that chemotherapy is able to enhance the tumor’s susceptibility to immunotherapy [32]. The NICITA study (NCT04177953) is a prospective, 1:1 randomized, open-label, multicenter phase II clinical trial. In this study, 92 patients who have already undergone surgery will be randomized in arm A and arm B and treated with the platinum-based chemotherapy with pemetrexed for a maximum of four cycles every four weeks, while patients in arm B will also receive 400 mg of nivolumab for ≤ four cycles and nivolumab alone will be administrated as maintenance therapy. The primary end point for this study is the time-to-next-treatment (TNT), which is the time from randomization until the initiation of any treatment because of disease progression. The association effect may be enhanced when chemotherapy is administrated during surgery (intraoperative hyperthermic chemotherapy). The study is currently enrolling patients [33].

For patients that are not eligible for MPM surgery, combination of nivolumab and chemotherapy is being evaluated as a first line treatment. JME-01 (UMIN000030892) is a phase II, single-arm, prospective, non-randomized, non-comparative, open-label, multicenter trial. Patients have been treated with cisplatin (75 mg/m^2^), pemetrexed (500 mg/m^2^) and nivolumab (360 mg) intravenously every three weeks for a total of four to six cycles. If patients have not progressed, maintenance therapy with nivolumab was administrated. Study primary end point is the ORR, while secondary end points are overall survival and PFS [32]. The study commenced in 2018 and recruited 18 participants: To September 2020 the ORR rate was 77.8%, while PFS and OS were 80 and 20.8 months, respectively [34].

Pembrolizumab is another anti-PD-1 human monoclonal antibody whose potential clinical efficacy for MPM treatment has been investigated in several studies. Results from the ETOP PROMISE-meso, (NCT02991482), the first randomized study evaluating the efficacy of pembrolizumab vs. standard chemotherapeutic agents in patients with recurrent MPM were presented at ESMO congress 2019. This open-label phase III trial, not limited by tumor PD-L1 expression, investigated the efficacy of pembrolizumab treatment (200 mg every three weeks) against the standard chemotherapy treatment (gemcitabine or vinorelbine) in relapsed MPM patients. The primary endpoint, PFS from 3.5 months to six months, was not met with a reported median PFS of 2.5 months vs. 3.4 months in the chemotherapy arm. Notably, the response rate (RR) was 22% in the pembrolizumab arm vs. 6% in the chemo arm, and median OS was 10.7 months vs. 11.7 for chemotherapy. Despite 45 chemotherapy patients crossed over to pembrolizumab, accounting for crossover yielded similar OS results. Adverse effects were experienced by 19% of pembrolizumab patients vs. 24% of chemotherapy patients, most commonly fatigue (19%) in the pembrolizumab arm vs. nausea (27%) and fatigue (31%) registered in the chemotherapy arm (Pr 2019). Overall, pembrolizumab did not prove to be superior to chemotherapy [35].

A non-randomized, open-label, phase Ib trial, the KEYNOTE-028 (NCT02054806), firstly investigated the anti-tumor efficacy of pembrolizumab in 25 PD-L1 positive malignant non-responder mesothelioma patients receiving 10 mg/kg of pembrolizumab every two weeks for up to two years or until progression or toxicity. The study is still ongoing but not recruiting, and to date 20% of patients had PR with a median response duration of 12 months [36]. The clinical benefit showed by these preliminary data led several phase II trials to start. KEYNOTE-158 (NCT02628067) is an ongoing phase II trial designed to research biomarkers predictive of response to pembrolizumab in several advanced solid tumors, including MPM. Another trial (KEYNOTE-139, NCT02399371) is assessing the activity of pembrolizumab as a second line therapy for advanced MPM and a third phase II active-comparator trial (NCT02784171) will explore the efficacy of first line therapy with pembrolizumab versus either cisplatin-pemetrexed or pembrolizumab-cisplatin-pemetrexed combination for advanced MPM. In all three studies, pembrolizumab dose is fixed at 200 mg once every three weeks.

Other studies were designed to assess the efficacy of PD-L1 inhibition. To this regard, the human IgG1 antibody avelumab targeting specifically PD-L1 was tested for MPM treatment in the phase Ib, open-label JAVELIN study (NCT01772004). The study enrolled 53 with unresectable pleural o peritoneal mesothelioma patients who had previously received a systemic treatment including pemetrexed and a platinum-based agent. Avelumab showed clinical anti-tumor efficacy in addition to a good safety profile: The confirmed ORR rate was 9%, with complete response in one patient and partial in the others, while DCR was 58%, PFS 17.4% and the median duration of response was 15.2 months. Overall toxicities were manageable, mostly grade 1 or 2 following the first or second infusion and only 9% of patients reported grade 3 or more treated-related adverse effects [37].

Another anti-PD-L1 agent that is being tested in several phase II and III clinical trials in various types of solid tumors is durvalumab. The DIADEM study (NCT04115111) is a phase II study designed to assess the activity and safety of this agent in monotherapy as a second line treatment in advanced pre-treated MPM. The 57 patients enrolled between October 2018 and May 2019 received durvalumab every four weeks until evidence of progression or inacceptable toxicity. The primary endpoint was the proportion of patients alive and free from progression at 16 weeks from the start of treatment. Secondary outcomes were PFS, OS, ORR together with the tumor growth index, the evidence of number of adverse events and the evaluation of PD-L1 expression and T lymphocytes infiltrations in tumor samples. The study is completed but no results have been posted yet.

The NCT0307527 trial was designed to assess the efficacy of the combined inhibition of PD-1/PD-L1 and CTLA-4 by associating durvalumab and tremelimumab. Unfortunately, the study was suspended because criteria for second stage were not met at time of interim analysis.

Efficacy of durvalumab is also being evaluated in combination with standard chemotherapy in PrE505 study (NCT02899195), an open-label, single arm phase II clinical trial. As of June 2018, the study enrolled 55 previously untreated patients with unresectable mesothelioma who were treated with the combination of durvalumab (1.120 mg) with cisplatin (500 mg/m^2^) and pemetrexed (75 mg/m^2^), every three weeks for up to six cycles. Subsequently, patients who had PR or SD were treated with a maintenance therapy with durvalumab for a maximum of 12 months. As of March 2020, the study met its primary endpoint with a median OS of 20.4 months compared to the control of 12.1 months. PFS at 6 months in patients treated with the combination was 69.1% and there was no significant correlation between tumor expression of PD-L1 or tumor mutational burden and PFS. To conclude, the combination of durvalumab and standard chemotherapy gave promising results in OS. This led to the phase III PrE506/DREAM3R study (NCT04334759), which again aims to investigate the efficacy of adding durvalumab to standard chemotherapy to improve OS in MPM patients. To date, March 2021, the study is still recruiting in the United States and Australia [38].

A novel approach to the immune checkpoint blockade is represented by the bispecific antibody AK104: it consists of a tetravalent antibody designed based on the Akeso Tetrabody platform (AACR, 2018), which targets both and concurrently PD-1 and CTLA-4. Notably, AK104 resulted to bind PD-1 and CTLA-4 with a greater avidity compared to the combination of single target antibodies. Moreover, dual blockade with such combination is limited by high rates of toxicity, while AK104 is reported to have greater safety. AK104 is now being tested in patients with relapsed or refractory mesothelioma (NCT03261011). Initial data were presented at ESMO 2020. The preliminary results suggest that AK104 is well tolerated and may represent an antitumor agent against mesothelioma, with an ORR of 15.4%, DCR of 84.6% and with seven patients showing tumor shrinkage [39]. However, further studies are required to evaluate AK104 efficacy against MPM.

**Table 1:** Clinical trials with anti-CTLA-4, anti-PD-1/PD-L1 and chemotherapy in MPM http://clinicaltrials.gov/ (accessed on 28 April 2021).

NCT Number	TRIAL Name	Treatment	Phase	Primary Endpoints	Therapy Setting	Status	Ref
**CTLA-4**							
NCT01649024	MESOT-TREM 2008	Tremelimumab	II	ORR	At least II line	Completed	[20]
NCT01655888	MESOT-TREM 2012	Tremelimumab	II	ORR	At least II line	Completed	[21]
NCT01843374	DETERMINE	Tremelimumab vs. Placebo	IIb	OS	At least II line	Completed	[22]
**PD-L1**							
NCT02497508	NivoMes	Nivolumab	II	DCR	At least II line	Completed	[24]
JapicCTI-163247	MERIT	Nivolumab	II	ORR	At least II line	-	[25]
NCT02497508	MAPS2	Nivolumab vs. Nivolumab + Ipilimumab	II	DCR	At least II line	Completed	[26]
NCT03048474	INITIATE	Nivolumab + Ipilimumab	II	DCR	At least II line	Completed	[27]
NCT03918252	-	Nivolumab + Ipilimumab	I/II	Safety Feasibility	Neoadjuvant	Recruiting	-
NCT03063450	CONFIRM	Nivolumab vs. Placebo	III	OS PFS	At least II line	Active, not recruiting	[30]
NCT02054806	KEYNOTE-028	Pembrolizumab	Ib	ORR	Any	Active, not recruiting	[36]
NCT02628067	KEYNOTE-158	Pembrolizumab	II	ORR	At least II line	Recruiting	-
NCT02399371	KEYNOTE-139	Pembrolizumab	II	PD-L1 cutoff	At least II line	Active, not recruiting	-
NCT01772004	JAVELIN	Avelumab	Ib	DLT Best OS	At least II line	Completed	[37]
NCT04115111	DIADEM	Durvalumab	II	Proportion of survived patients at 16w	At least II line	Completed	-
NCT02899195	PrE505	Durvalumab	II	OS	First line	Active, not recruiting	[38]
NCT03261011	-	AK104	I	AEs DLT	At least II line	-	-
**IC/CT**							
NCT02899299	CheckMate 743	Nivolumab + Ipilimumab vs. CT	III	OS	First line	Active, not recruiting	[28]
UMIN000030892	JME-01	CT + Nivolumab	II	ORR	First line	Active	[34]
NCT02991482	PROMISE-Meso	Pembrolizumab vs. CT	III	PFS	At least II line	Active, not recruiting	[35]
NCT02784171	-	Pembrolizumab vs. Pembrolizumab + CT	II/III	II: PFS III:OS	First line	Active, not recruiting	-
NCT04334759	PrE506/ DREAM3R	Durvalumab + CT vs. CT	III	OS	First line	Recruiting	-

AEs, adverse events; CT, chemotherapy; DCR, disease control rate; DLT, dose limiting toxicity; MTD, maximum tolerated dose; IC, immune checkpoint; ORR, overall response rate; OS, overall survival; PAD, pharmacologically active dose; PFS, progression free survival; TD, tolerated dose; TEAE, treatment-emergent adverse event; TNT, time to next treatment.

### 2.3. LAG-3, TIM-3 and VISTA

Lymphocyte activation gene 3 (LAG-3, CD223) and T cell immunoglobulin 3 (TIM-3) are cell surface molecules belonging to the Ig superfamily expressed on activated T cells, and on other cells involved in the immune response. In the last few years, an altered expression of these molecule on TILs has been described in a number of solid tumors including MPM.

LAG-3, firstly identified on activated human NK and T cells [40], is a cell surface receptor with structural similarities to CD4, which negatively regulates antigen-specific T cell responses.

In addition to activated CD4^+^, CD8^+^ effector T cells and NK cells [41,42], LAG-3 expression has been detected on B cells [43] and plasmacytoid dendritic cells (DCs) [44]. Under physiological conditions, LAG-3 acts as a negative regulator of recently activated and chronically stimulated exhausted T cells, showing an important role in the homeostatic expansion of T cells [45,46], whereas the role of LAG-3 on NK cells, B cells, and DCs is not yet fully understood [43,47]. LAG-3 is also expressed on CD4^+^Foxp3^+^ Treg_s_ having immunosuppressive function: [48], even if its role on Treg_s_ has not been well characterized yet [49].

LAG-3 has been proposed to bind to MHCII with higher affinity than CD4 and therefore to inhibit T cell activation, [49]. During the years, several additional ligands of LAG-3 have been found, because of the capability of this protein to interact with cells that do not express MHCII. Galectin-3, a soluble lectin, and liver sinusoidal endothelial cell lectin (LSECtin), a cell surface lectin expressed in the liver, are both alternative ligands for LAG-3 [50,51].

Over-expression of LAG-3 has been found on TILs in various human tumors, including MPM, and is often correlated with general T cell dysfunction across several human malignancies [52]. The frequent co-expression of LAG-3 with other immune checkpoint inhibitors such as PD1, PD-L1, CTLA-4, and TIM-3 [47,52] strongly indicates that LAG-3 might contribute to immune escape mechanisms in cancer. In addition, LAG-3 has been detected in the pleural and ascites fluids of MPM patients [10,53]. Accordingly with these observations, the LAG-3 immune checkpoint has been identified as a potential target to improve productive tumor-specific T cell immunity, and various LAG-3 inhibitors have been developed and their efficacy evaluated both in preclinical models of MPM and in clinical trials. In particular, targeting the LAG-3 pathway has been associated with anti-tumor activity in preclinical models, providing further rationale for pharmacologic modulation of the LAG-3 axis in cancer patients [49]. Very recently a combination study showed that simultaneous PD-1 and LAG-3 blockade exerted an increased effect in the maintenance of T cell activity in vitro, evaluated as cytokines release, and resulted in delayed tumor growth and survival benefit in vivo in a syngenic mouse model of MPM respect to single ICI treatment [54].

In the clinical setting, LAG-3 inhibitors are currently under evaluation, also in combination with pembrolizumab or nivolumab, for the treatment of different types of human tumors [47,52] and at the moment, two clinical trials are ongoing in MPM patients.

INCAGN02385 is an antagonist antibody targeting LAG-3 under evaluation in a phase I trial in patients with selected advanced malignancies including MPM (NCT03538028). The purpose of this study was to determine the safety, tolerability, and preliminary efficacy of the compound. The study was completed in October 2020; however, no results are available till now (Table 2).

Ieramilimab (LAG525) is another monoclonal antibody against LAG-3 and was under evaluation in a Phase I/II clinical trial as single agent or in combination with the anti-PD-1 antibody PDR001 in patients with advanced malignancies who had disease progression following their last prior therapy, including mesothelioma in group 4 (NCT02460224 A). The purposes of the study were the evaluation of the incidence of dose limiting toxicities (DLTs) and the ORR per RECIST V1.1. The study was completed but the results are not available yet.

TIM-3 belongs to the *TIM* gene family on chromosome 5, together with TIM-1 e TIM-4, and was first identified as a specific marker for CD4^+^ Th1 and CD8^+^ T cytotoxic cells [55,56]. TIM-3 is also expressed on NK cells, CD4^+^ Foxp3^+^ Treg_s_, B cells, DCs, monocytes, and macrophages [57,58]. Under physiological conditions, TIM-3 is a negative regulator of CD4^+^ Th1 and CD8^+^ T cytotoxic cells activity [58]; similarly, it has been described as a negative regulator of DCs and macrophages function [55]. The suppressive function of TIM-3 has been reported also for CD4^+^Foxp3^+^ Treg_s_ [58]. Moreover, TIM-3^+^ Treg_s_ showed increased expression of other inhibitory receptors, such as CTLA-4, LAG-3, and PD-1 and high secretion of suppressive cytokines, such as IL-10 and TGF-β [59,60]. NK cells also express high levels of TIM-3, but the role of this protein in the regulation of NK activity is still controversial [58].

So far, four TIM-3 ligands have been identified: C-Type lectin galectin-9 (galectin-9), carcinoembryonic antigen cell adhesion molecule 1 (Ceacam1), high-mobility group protein B1 (HMGB1) and phosphatidyl-serine (PtdSers). Galectin-9 interacts with the TIM-3 N-terminal Ig variable region (IgV)-like domain, promoting cell death [61,62]. Ceacam1 is co-expressed with TIM-3 on T cells, acting as a negative regulator of T cells activities [63]. HMGB1, a soluble ligand, binds TIM-3 showing inhibitory functions in DCs [64]. PtdSers interacts with TIM-3^+^ phagocytic cells to promote uptake of apoptotic cells [65,66].

Many studies have shown that TIM-3 is highly expressed on TILs in different types of human tumors, and its expression is related with cancer severity and poor prognosis [67,68]. Moreover, it has been demonstrated that TIM-3 is frequently co-expressed with PD-1 on TILs in mice bearing solid tumors and co-blockade of both proteins showed a recovery of T cell function and a tumor regression in 50% of mice [69]. Similarly, a study performed on mice with disseminated acute myelogenous leukemia confirmed that combined PD-1 and TIM-3 blockade was more effective than single treatment [70].

The expression of TIM-3, together with other immune checkpoints (PD-L1, PD-1, LAG-3) has been evaluated in vivo by flow cytometry in 6 pleural and 5 ascites fluid samples from chemotherapy-treated MPM patients. In both fluids’ types, CD4^+^ T cells, CD8^+^ T cells and NK cells expressed PD-1, LAG-3, and TIM-3, even if with some differences depending on the cell type and on the immune checkpoint molecule, suggesting LAG-3 and TIM-3 as possible novel therapeutic targets for the treatment of MPM [53]. Another study performed on 54 tissue sections from MPM patients (40 samples at time of diagnosis and 14 samples chemotherapy-treated) investigated the immune checkpoint expression profile in MPM by immunohistochemistry [71]. TIM-3 was expressed on TILs of both untreated and treated samples (40% vs. 29%, respectively). Moreover, data showed a strong correlation between TIM-3^+^ TILs and PD-L1^+^ TILs in the stroma (RR = 0.48; *p* < 0.001). Interestingly, TIM-3 was also found on tumor cells of both untreated and treated samples (40% and 36%, respectively). These findings support the rationale for targeting TIM-3 as a therapeutic strategy for MPM treatment.

A recent study investigated immunological markers using multiparametric flow cytometry in order to discriminate MPM from pleuritic and from pleural metastases. The samples consisted of pleural fluids from nonmalignant pleuritis (63), MPM (49) and pleural metastases (32), and of tissue biopsies from nonmalignant pleuritis (16), MPM (33) and pleural metastases (5). The results showed that PD-1, LAG-3, and TIM-3 were highly expressed on CD4^+^ T cells and CD8^+^ T cells of MPM samples compared to T cells of pleuritis and pleural metastases samples. This study identified potential prognostic immune biomarkers that could be used for a rational and personalized immunotherapy in MPM patients [10].

Since 2016, many TIM-3 antagonist monoclonal antibodies, used as single agents or in combination with antibodies targeting other immune checkpoint inhibitors, entered several clinical trials for the treatment of advanced solid tumors and hematological malignancies [72].

The monoclonal antibody INCAGN02390 against TIM-3 is now under evaluation in a clinical phase I, open-label, dose-escalation clinical trial (NCT03652077), with the purpose to determine its safety, tolerability, and preliminary efficacy in patients with select advanced malignancies, including MPM.

VISTA, also known as C10orf54, differentiation of ESC-1 (Dies1), platelet receptor Gi24 precursor or PD1 homolog (PD1H), is an immune checkpoint protein, firstly identified in 2011 in murine models [73,74,75]. VISTA belongs to the B7 family and shares 22% of the sequence with PD-L1, which represents its closest homolog within the B7 family [76,77]. VISTA is a negative immune checkpoint regulator that induces immunosuppressive activities on T cells, showing a critical role in the context of autoimmunity, inflammation, and anti-tumor immunity [73,74,76].

VISTA is mainly expressed on hematopoietic cells [73]. On myeloid cells, VISTA is constitutively expressed on neutrophils, monocytes, macrophages, basophils, and DCs [78]. On lymphocytes, VISTA is mainly expressed on naïve CD4^+^ T cells and on FoxP3^+^ Treg_s_. On the contrary, CD8^+^ T cells, NK cells, and thymocytes have low levels of VISTA. Although B cells do not express VISTA, this protein seems to be highly expressed on plasma cells [78].

Whether VISTA acts as a receptor or a ligand, or it could have both functions depending on the type of cell, has been unclear until recent studies have proposed VISTA as a receptor, and VSIG3 (or IgSF11) and P-selectin glycoprotein ligand 1 (PSGL-1) as possible ligands [79,80].

VISTA expression was recently investigated in human cancers. So far, many studies have shown that VISTA is expressed on both cancer cells and TILs in various types of human cancer, such as oral squamous cell carcinoma [81], gastric cancer [82], colorectal carcinoma [83], ovarian cancer [84], hepatocellular carcinoma [85] and NSCLC [86]. On the other hand, in primary cutaneous melanoma [87] and in pancreatic cancer [88], VISTA is expressed only on tumor-infiltrating inflammatory cells.

In 2018, a genomic study on 74 MPM samples, part of The Cancer Genome Atlas, demonstrated for the first time that VISTA is also expressed in MPM [11]. The data showed that the epithelioid subtype of MPM had the highest levels of VISTA mRNA. Then, immunohistochemistry staining for VISTA, performed on two epithelioid MPM samples, revealed that this protein was expressed on cancer cells, TILs, and normal mesothelium. Based on these results, the authors speculated that normal mesothelium expressed VISTA due to its APC property and the fact that also epithelioid MPM expressed this protein suggested that APC properties are conserved during MPM cancerogenesis [11].

In another study, the levels of VISTA and PD-L1 were investigated by immunohistochemistry staining on 37 MPM tissue samples, in order to evaluate the relationship between these two immune checkpoint inhibitors in MPM patients. Of the 37 total tissue samples, only 26 were tested for both VISTA and PD-L1. Data showed that VISTA was expressed ≥1% in 25 (96%) and ≥50% in 22 (84%) cases, whereas PD-L1 was expressed ≥1% in 11 (42%) and ≥50% in two (8%) cases, suggesting no evident correlation between VISTA and PD-L1 expression in MPM [89]. In 2020, Muller and collaborators, based on the results of The Cancer Genome Atlas study, performed immunohistochemistry staining for VISTA and PD-L1 in 319 pleural MPM tissue samples (254 epithelioid, 24 biphasic, and 41 sarcomatoid) and 10 tissue samples of normal pleura. The data obtained showed that MPM samples expressed VISTA and PD-L1, respectively 88% and 33% in epithelioid, 90% and 43% in biphasic, and 42% and 75% in sarcomatoid histotype. Epithelioid MPM samples had a median VISTA score higher than biphasic and sarcomatoid (50% vs. 20% and 0% respectively, (*p* < 0.001)). On the other hand, sarcomatoid MPM samples showed a median PD-L1 score higher than biphasic and epithelioid (20% vs. 0% and 0% respectively, (*p* < 0.001)). All samples of non-neoplastic mesothelium tissue expressed VISTA, thus confirming the abovementioned studies of Hmeljak [90]. Taken together, these findings suggest VISTA blocking as a novel therapeutic strategy for the treatment of MPM.

CA-170 is an oral small molecule that inhibits PD-L1, PD-L2, and VISTA immune checkpoints. Previous in vivo studies showed that CA-170 promoted activation and proliferation of a subset of T cells whose activity was impaired by PD-L1 or VISTA [91], and inhibited tumor growth in multiple mouse syngeneic cancer models [92].

This small molecule entered a Phase Ib trial (NCT02812875), designed to investigate its safety, pharmacokinetic, pharmacodynamic, and clinical effects in patients with advanced tumors and lymphomas known to have a high VISTA expression, including MPM patients who received at least one chemotherapy treatment prior to enrolment. CA-170 evidenced a safe toxicological profile and favorable clinical PK; however, the drug showed modest anti-tumor activity, and only one patient on 12 enrolled remained on the study treatment for more than 21 weeks with stable disease (SD) [93].

**Table 2:** Clinical trials with anti-LAG-3, anti-TIM-3, anti-VISTA http://clinicaltrials.gov/ (accessed on 28 April 2021).

NCT Number	Treatment	Phase	Primary Endpoints	Therapy Setting	Status	Ref
**LAG-3**						
NCT03538028	INCAGN02385	I	TEAEs	Any	Completed	-
NCT02460224	Ieramilimab	I/II	DLTs ORR	Any	Completed	-
**TIM-3**						
NCT03652077	INCAGN02390	I	TEAEs MTD or PAD	Any	Active, not recruiting	-
**VISTA**						
NCT02812875	CA-170	Ib	DLT MTD Recommended phase 2 dose	Any	Completed	-

DLT, dose limiting toxicity; MTD, maximum tolerated dose; ORR, overall response rate; PAD, pharmacologically active dose; TEAE, treatment-emergent adverse event.

## 3. CAR-T Cell Therapy

CAR-T cell therapy is a promising therapeutic approach within the field of cancer immunotherapy, for the treatment of various types of hematological malignancies and solid tumors, including MPM [94,95]. CAR-T cell therapy consists of taking lymphocytes from a patient, equipped them with chimeric antigen receptors (CARs) designed to bind tumor-associated antigens (TAAs), expanded them ex vivo, and finally re-infused the cells into the same patient, either systemically or regionally, in order to induce the immune system to recognize and destroy cancer cells [94,95]. So far, four generations of CARs have been created to enhance anti-tumor efficacy. “First-generation” CARs are composed of a single-chain variable antibody fragment (scFv) fused to CD3ζ intracellular domain [96]. This type of CAR shows cytotoxicity in vitro and poor efficacy in vivo, suggesting the need to overcome these features [96,97]. “Second-generation” CARs have scFv fused to CD3ζ with co-stimulatory domains including CD28 and 4-1BB, and show greater anti-tumor efficacy [94]. “Third-generation” CARs have CD3ζ with two co-stimulatory domains [96]. Finally, a “fourth-generation” CAR-T cells, called TRUCK_s_ (T cells redirected for antigen-unrestricted cytokine-initiated killing) were designed also to release a transgenic cytokine in the tumor stroma in order to induce an anti-tumor immune response [98]. Based on encouraging results obtained both in vitro and in murine models, there are currently some ongoing phase I clinical trials using TRUCKs for the treatment of solid tumors [98].

The identification of TAAs plays a crucial role for CAR-T cell therapy. In fact, these antigens must be overexpressed on cancer cells and, at the same time, they must be poorly expressed on normal tissues. Over the years, some possible targets have been identified for the MPM CAR-T cell therapy and both preclinical and clinical trials have been developed in order to investigate this therapeutic approach for the treatment of MPM.

The cell surface proteoglycan chondroitin sulfate proteoglycan 4 (CSPG4) is over-expressed on MPM cell lines and on MPM human biopsies [99], suggesting it as a target for MPM CAR-T cell therapy. Indeed, a preclinical study using CSPG4-targeted CAR expressed on T cells from MPM patients showed that patient-derived CSPG4 CAR-T cells promoted cytokine release and caused cytotoxicity in co-culture system with MPM cell lines [100].

Other appealing TAAs for MPM CAR-T cell therapy are the members of ErbB family receptors. Indeed, high levels of both EGFR and ErbB4 (79.2% and 49.0%, respectively) were found by immunohistochemical analysis on human MPM tissue samples, thus prompting the production of a panErbB-targeted CAR expressed on T cells from MPM patients. These patient derived CAR-T cells demonstrated cytotoxicity in vitro in a panel of MPM cell lines, and tumor regression in MPM xenografts [101].

MET receptor tyrosine kinase represents another possible target for the MPM CAR-T cell therapy, because this protein is overexpressed in MPM [102,103,104,105]. Based on this, a pre-clinical study investigated the efficacy of MET-specific CARs in MPM, showing in vitro the elimination of MET-expressing human MPM cell lines and in vivo the regression of MPM xenografts [106].

Mesothelin is a cell surface glycoprotein that represents an attractive target for the MPM CAR-T cell therapy. Indeed, this protein shares an important role in the processes of malignant transformation and metastasis and is overexpressed in various types of solid tumors, including MPM, in particular in the epithelioid histotype [107,108,109], whereas it is expressed at very low levels on normal mesothelial cells. Anti-mesothelin second-generation CARs were investigated for the first time in a phase I clinical trial on MPM patients in order to evaluate the safety of this therapeutic approach (NCT01355965); although this treatment showed moderate clinical responses, no patient showed toxicity in normal tissue due to anti-mesothelin CAR_s_ infusions, demonstrating the safety of this therapeutic strategy (Table 3) [110]. Subsequently, anti-mesothelin second-generation CAR-T cells used at two different concentrations with and without cyclophosphamide were investigated in a safety phase I clinical trial in different types of solid tumors, including MPM, (NCT02159716). This study demonstrated the safety of the treatment and the presence of CAR-T cells in patients’ blood for at least 30 days; however, no clinical response was achieved [94]. A third on-going phase I clinical study was designed to investigate the safety and feasibility of intravenous or intrapleural administered lentiviral transduced huCART-mesothelin cells (supposed to be more effective in comparison with the previous used second-generation CAR-T cells) administered with or without cyclophosphamide in solid tumors, including histologically confirmed epithelioid MPM (NCT03054298). The study is ongoing, and data are not yet available. Another on-going phase I/II clinical trial aims to evaluate safety of different doses of mesothelin-targeted T cells administered into the pleura in patients with malignant pleura diseases. This clinical investigation also plans to evaluate the safety of the association of the anti-mesothelin CARs in combination with pembrolizumab for MPM patients (NCT02414269). Preliminary results from 25 patients affected by MPM were presented at the 2019 American Society of Clinical Oncology (ASCO) meeting and showed encouraging results. Indeed, the patients who received CAR-T cell therapy, cyclophosphamide, and at least three doses of pembrolizumab achieved either ORR (36.8%) and a DCR (57.8%). Moreover, CAR-T cells persisted in the pleural fluid of responsive patients for up to 42 weeks [111].

Fibroblast activation protein (FAP), a transmembrane serine protease, is overexpressed in the cancer-associated stromal cells of all three MPM subtypes [112], suggesting it as a possible target for the MPM CAR-T cell therapy. Based on encouraging preclinical studies [113], a phase I clinical trial was designed to investigate the safety of a fixed single dose of FAP-targeted CAR-T cells administered into the pleura of MPM patients (NCT01722149). Preliminary results showed a good tolerance of the treatment and the persistence of CAR-T cells following inoculation; however, due to the trial design and the small number of patients involved, it was not possible to evaluate the impact of this treatment on patient outcome [114].

**Table 3:** Clinical trials with CAR-T cells and cancer vaccines in MPM http://clinicaltrials.gov/ (accessed on 28 April 2021).

NCT Number	Treatment	Phase	Primary Endpoints	Therapy Setting	Status	Ref
**CAR-T**						
NCT02159716	CART-meso	I	AEs	-	Completed	[94]
NCT01355965	Autologous T-cells	I	AEs	-	Completed	[110]
						
NCT03054298	huCART-meso cells	I	Treatment related AEs	-	Recruiting	
NCT02414269	iCasp9M28z T cell infusions; iCasp9M28z T cell infusions + Pembrolizumab	I/II	I: AEs II: clinical benefit rate	At least II line	Recruiting	[111]
NCT01722149	Adoptive transfer of re-directed T-cells	I	Safety	-	Completed	[114]
**Vaccines**						
NCT00280982	Tumor lysate loaded autologous dendritic cells	I	Safety Tolerability	-	Completed	[115]
NCT01241682	Dendritic cells + CTX	I	Number of cytotoxic and regulatory T-cells in patients’ blood	-	Completed	[116]
NCT02395679	MesoCancerVac	I	TD		-	[117]
NCT03610360	MesoPher	II/III	OS	At least II line	Recruiting	[118]
NCT01503177	oncolytic measles virus encoding thyroidal sodium iodide symporter	I	AEs	-	Completed	-
NCT01265433	WT-1-vaccine Montanide + GM-CSF	II	PFS 1 year	At least II line	Completed	[119]
NCT04040231	Galinpepimut-S + Nivolumab + GM-CSF	I	MTD	-	Recruiting	-
NCT01675765	CRS-207	I	AEs Induction of response to mesothelin	-	Completed	[120]

AEs, adverse events; MTD, maximum tolerated dose; OS, overall survival; PFS, progression free survival; TD, tolerated dose.

## 4. Cancer Vaccines

Therapeutic cancer vaccines represent one of the most studied approach of immunotherapy and aim to stimulate an anti-tumor immune response in cancer patients [121]. DCs vaccines, genetic vaccines, and peptide vaccines are considered the three main types of this anti-cancer strategy based on TAAs.

Based on their function of “professional APCs”, DCs are used for an anti-tumor immunotherapeutic approach called DC-based immunotherapy in order to activate a robust anti-tumor immune response aimed to eliminate cancer cells [122]. The first step of this strategy is to generate DCs using ex vivo or in vivo methods. The most common ex vivo method is characterized by culturing monocytes in vitro, using growth factors and cytokines, to obtain the differentiation in DCs [123]. Another ex vivo method involves the use of CD34^+^ progenitors, mobilized from the bone marrow, which are differentiated into DCs by culturing in vitro, using growth factors and cytokines [124]. Finally, the in vivo method consists in the expansion of circulating DCs, after administration of growth factors [125]. Once DCs are obtained, the second step is loading these cells with TAAs using different strategies. In many clinical trials, DCs were loaded with tumor cell lysate, showing no toxicity and great anti-tumor immune response in most cases [126]. In other clinical studies, DCs were pulsed with TAAs resulting in an effective therapeutic response [126]. The use of viral vectors or mRNA encoding for TAAs represent an attractive option for loading TAAs on DCs [122]. The last step is the administration of DC vaccines through various routes, i.e., intra-venous, subcutaneous, intra-lymphatic, intra-dermal, or intra-nodal.

Since the mid-1990s, many clinical trials were designed to assess safety and feasibility of DC-based cancer vaccines for the treatment of various types of hematological malignancies and solid tumors, including MPM. Encouraging results for the use of DCs vaccines came firstly from preclinical studies in mouse models of MPM. Murine DCs were pulsed with tumor cell line lysates and injected in syngeneic mice either before, at the day of or after tumor implantation, demonstrating that mice receiving DCs vaccine before tumor implantation had protective anti-tumor immunity due to a strong tumor-specific cytotoxic T lymphocyte (CTL) response; when DCs were administered after tumor implantation as a therapeutic setting, the most beneficial effects were obtained at early stages of tumor development, being mesothelioma outgrowth prevented in this setting [127].

On the basis of these findings, in 2010 the same group proposed a phase I clinical trial using DCs vaccine for the treatment of 10 MPM patients (NCT00280982). For this study, autologous monocyte-derived DCs loaded with autologous tumor cell lysates were administered in MPM patients pretreated with chemotherapy, demonstrating the activation of an effective anti-tumor immune response and the safety and feasibility of the treatment; moreover, three patients showed PR in the first eight weeks after DCs vaccination [115].

Few years later, another phase I clinical trial was designed to evaluate the safety and toxicity of the administration of autologous DCs pulsed with autologous tumor lysate combined with low-dose cyclophosphamide to increase the anti-tumor activity by inhibiting Treg_s_ for the treatment of 10 MPM patients pretreated with chemotherapy (NCT01241682). The data from this study indicated that the treatment was safe, due to the absence of adverse effects, and highlighted an anti-tumor effect with seven out of 10 patients having a survival of greater than or equal to 24 months and two patients alive after 50 and 66 months [116]. More recently, another study (NCT02395679), on the basis of promising results obtained on MPM mouse models, aimed to investigate the safety and feasibility of autologous monocyte-DCs pulsed with allogenic tumor cell line lysates (obtained from five mesothelioma cell lines) in 9 MPM patients, demonstrating no dose-limiting toxicity and induction of immune anti-tumor response [117]. In particular, two patients showed PR and all patients reached DC, with a median OS of 22.8 months. On the basis of these encouraging results, an ongoing phase II/III trial (NCT03610360), having OS as primary end point, is evaluating DCs loaded with allogeneic tumor cell lysates as a maintenance therapy after chemotherapy (DENIM trial) [118].

Genetic vaccines include DNA vaccines, which are bacterial plasmids encoding for TAAs [128,129], RNA vaccines [130,131], and viral-based vaccines, which use viruses to stimulate an antigen-specific anti-tumor immune response. The latter therapeutic approach, called also oncolytic viral immunotherapy, uses nonpathogenic oncolytic viruses to infect and lyse cancer cells and to stimulate a robust immune anti-tumor response, either innate or adaptive, resulting in an alternative therapeutic approach for many cancers, including MPM [132,133].

Various types of oncolytic viruses have been tested in many pre-clinical and clinical studies for the treatment of MPM [132,133]. Among them, adenovirus represents the most studied vector for MPM viral immunotherapy [132]. A recent preclinical study used the mutant adenoviral dl922-947, an engineered adenovirus bearing a deletion in the E1A-Conserved Region 2, which allowed selective infection and replication in cancer cells with a defective retinoblastoma (RB) pathway, including MPM cells. The authors demonstrated that dl922-947 exerted cytotoxic effects on MPM cell lines and anti-tumor effects in a MPM murine xenograft model, resulting in a decrease in tumor growth [134]. Such an approach might be relevant for MPM treatment due to the highly frequent loss of the *CDKN2A* locus encoding the RB upstream regulator p16 found in this type of tumor [11]. Similarly, a recent in vitro study evaluated the anti-tumor activity of oncolytic Schwarz strain of measles virus in MPM cell lines and showed that *CDKN2A* homozygous deletions are frequently associated with the homozygous deletions of type I interferon (IFN-I) genes, which are located in the same 9p21.3 chromosome region, thus rendering these cell lines more sensitive to measles virus infection [135]. It is worth of note that the loss of *BAP1* might be involved in the sensitivity to oncolytic virus therapy for MPM patients. In fact, a recent study suggested that MPM with *BAP1* loss may be resistant to oncolytic viral therapy, due to a significant increase in the IFN-I pathway [136]. Similarly, an in vitro study confirmed the correlation between *BAP1* loss and resistance to oncolytic measles virus therapy in MPM cell lines, even if under this condition, a role of IFN-I pathway was not demonstrated, probably due to the absence of the immune infiltrate [137]. Therefore, the identification of genetic status of *CDKN2A* and *BAP1* could be a screening tool to identify MPM patients suitable for oncolytic measles virus therapy.

Currently, a phase I clinical trial aims to investigate both therapeutic and adverse effects and the best dose of intrapleural oncolytic measles virus (MV-NIS virus) in 15 MPM patients (NCT01503177). The study is completed but no data are available till now.

Peptide vaccines represent a therapeutic anti-tumor approach consisting in the administration of TAAs peptides that are recognized by T cells, in order to stimulate an anti-tumor immune response in cancer patients [121]. The most important requirement for this approach is that the peptides are derived from antigens expressed only by cancer cells and not by normal tissues.

Wilms’ tumor 1 (WT1) is considered an attractive target for the development of peptide vaccines for the treatment of MPM. Indeed, a study showed that WT1 was expressed in 97% and 98% of epithelioid and non- epithelioid MPM cases, respectively, out of a total of 283 MPM patients [138]. The Wilms’ tumor gene (11p13), primarily associated with Wilms’ tumor, a pediatric kidney cancer [139], has been found expressed in many types of adult tumors, but is absent in healthy tissues [119]. Based on a previous study demonstrating activation of the immune response after administration of WT1 peptide vaccination in MPM patients [120], other clinical trials have recently been developed. In 2017, a phase II clinical trial (NCT01265433) showed that the analog WT1 peptide vaccine galinpepimut-S was safe and well-tolerated in MPM patients pretreated with multimodality therapy. Moreover, galinpepimut-S improved MPM patient outcome, showing median PFS and OS respectively 36% and 25% longer than PFS and OS of MPM patients treated with placebo [140].

Finally, another ongoing phase I clinical study (NCT04040231) aims to test the safety of administration of galinpepimut-S alone and in association with nivolumab in MPM patients who have received at least one prior cycle of pemetrexed-based chemotherapy.

Mesothelin is considered another suitable target for the treatment of MPM using different immunotherapeutic approaches, including peptide vaccines. In fact, a recent study shows that mesothelin is expressed in 93% of non-epithelioid MPM cases, confirming its over-expression in this rare tumor [138]. A recent phase I clinical trial (NCT01675765) aimed to investigate the safety and the anti-tumor immune response of the administration of cancer vaccine CRS-207 (an attenuated form of *Listeria monocytogenes* engineered to stimulate an immune response against mesothelin), with or without cyclophosphamide, followed by standard chemotherapy in MPM patients. The results showed that the administration of CRS-207 was safe, due to the absence of listeriosis, and was well tolerated in combination with cisplatin and pemetrexed. The study achieved encouraging results, with 89% of patients showing DC, 54% PR, and 29% SD, with a median PFS and OS of 7.5 and 14.7 months, respectively. Additionally, 31% of patients had tumor size reduction after treatment with CRS-207 followed by chemotherapy [141].

## 5. Conclusions

The clinical studies conducted in the last few years have failed to show an evident advantage of ICIs in comparison with the canonical chemotherapy treatment. Indeed, although some MPM patients had a good outcome and durable clinical benefit together with few adverse events, for the majority of patients there was no appreciable benefit and tumors underwent a rapid progression. In these studies, PD-L1 expression did not emerge as a biomarker of clinical response to ICIs treatment; therefore, there is an urgent need to identify predictive factors of response, in order to select those patients who may benefit the most. Among them, VISTA, LAG-3, TIM-3 might represent new immunological markers to be addressed. In particular, being VISTA mainly expressed by the epithelioid subtypes of MPM, anti-VISTA therapy could represent a target approach for this type of tumors. Moreover, other characteristic genomic alterations of MPM represent predictive biomarkers of response to immunotherapy. Indeed, as previously reported, *CDKN2A* loss often co-occurs with the deletion of *INF-I* genes, thus rendering the cells most sensitive to the therapy with oncolytic viruses, whereas *BAP1* mutations may impair the efficacy of this type of immunotherapy [136,142]. Other tumor suppressor genes, often altered in MPM patients, are *LATS1/2* [11,143], negative regulators of the Hippo-YAP pathway, whose deletion improved tumor immunogenicity together with an enhanced anti-tumor immune response [144]. Recently, it has been demonstrated that *LATS1/2* alterations could define a subset of MPM patients that might benefit of ICIs treatment. Indeed, MP patients harboring *LAST1/2* alterations showed an increased nuclear YAP activity, associated with high PD-L1 expression and infiltrative immune signature [145]. Additionally, the tumor suppressor gene *NF2* is often lost in MPM patients, and it has been correlated with compromised mediated immunity by B cells [145]. These findings indicate that frequently occurring MPM genetic alterations may have a role in tumor immunity and provide an early tool of biomarkers that may help to guide the management of immunotherapy in MPM patients [146].

In addition, it is worth noting that the majority of the clinical trials with ICIs have been initially conducted in the salvage setting as second or third line treatment in patients who had progressed after previous regimens based on chemotherapy, and the limited life span of these patients could explain the poor or in some cases absent benefit of such treatments.

These considerations are corroborated by the outcome of the CheckMate 743 trial, where the effect of nivolumab-ipilimumab double treatment was compared to cisplatin-pemetrexed in the front-line setting in unresectable MPM patients. A significant OS benefit for nivolumab-ipilimumab treatment was observed, with similar clinical effects in both epithelioid and non-epithelioid subtypes, whereas chemotherapy was markedly inferior in non-epithelioid subtypes.

Based on these encouraging results, a number of other phase III clinical trials are ongoing, evaluating ICIs in combination with chemotherapy in the first line setting treatment. For example, the BEAT-Meso phase III trial (NCT03762018) is evaluating the addition of atezolizumab to bevacizumab plus standard chemotherapy with carboplatin/pemetrexed until disease progression. A total of 320 participants from approximately 45 centers in Europe are expected to be included in the trial, whose primary endpoints are OS and PFS. The DREAM3R phase III trial (NCT04334759) is recruiting patients to evaluate the efficacy of durvalumab associated with cisplatin/pemetrexed as first line treatment in patients with advanced MPM. The trial expects to enroll 480 participants and the primary endpoint is OS. The abovementioned Canadian phase II/III trial NCT02784171 is evaluating the benefit of pembrolizumab combined with cisplatin-pemetrexed as first line treatment in patients with advanced MPM. Primary endpoints are PFS (phase II) and OS (phase III).

One of the hypotheses underlying these associations is that chemotherapy might increase the scarce tumor molecular burden (TMB) found in mesothelioma patients [147], therefore augmenting the efficacy of the simultaneous ICIs treatment. Indeed, the tumor neoantigen repertoire is essential for the activation and function of effector T cells.

Relevant for ICIs efficacy is also tumor T cell infiltration; “cold tumor”, characterized by a poor lymphocyte infiltration, are often less responsive to immunotherapy. Besides chemotherapy, other strategies might be exploited to increase the infiltrating population of immune cells, among them the use of the above-mentioned oncolytic viruses or the administration of immune-stimulating cytokines, such as IL-2 and GM-CSF. Moreover, radiotherapy is being investigated in two ongoing clinical trials (NCT02959463, NCT03399552) as an additional approach aimed at potentiating the anti-tumor immune response induced by PD-1 (pembrolizumab) or PD-L1 (avelumab) blockade. To improve the efficacy of ICIs, alternative combinatorial strategies are currently being evaluating, including combination with antiangiogenic agents (bevacizumab, ramucirumab, nintedanib), with the FAK inhibitor defactinib, or with mesothelin-targeted therapies, such as the immunotoxin LMB-100 and the antibody-drug conjugate anetumab ravtansine. These studies are more extensively described elsewhere [8,148].

Besides the use of ICIs, several other strategies are under evaluation to favor MPM immunomodulation, such as cellular therapies, based on the development of CAR-T cells. The advantage of specific MPM TAAs, such as mesothelin, together with the possibility to directly administer CAR-T cells intrapleurally, renders this approach promising, and the results obtained from the recent studies gave initial signals of efficacy and safety.

Another promising immunological approach is based on the use of cancer vaccines as the DCs therapy, and a phase II/III trial is actually ongoing (NCT03610360), using autologous DCs “labeled” in vitro with tumor lysate. In this field, another interesting approach is represented by the regional administration of oncolytic viruses, with the ongoing phase I/II clinical trials demonstrating some benefits and responses, even if additional investigations are warranted.

In conclusion, despite ICIs being a very promising treatment in many cancers, further studies are needed to confirm their efficacy in MPM.
